# Supplementary figures and images for: Cloning, expression, and molecular modification of glycoside hydrolase family 5 genes from Thermoascus aurantiacus
Source: PLoS One. 2023 Sep 15;18(9):e0285680. doi: 10.1371/journal.pone.0285680 (PMC10503741; doi:10.1371/journal.pone.0285680)

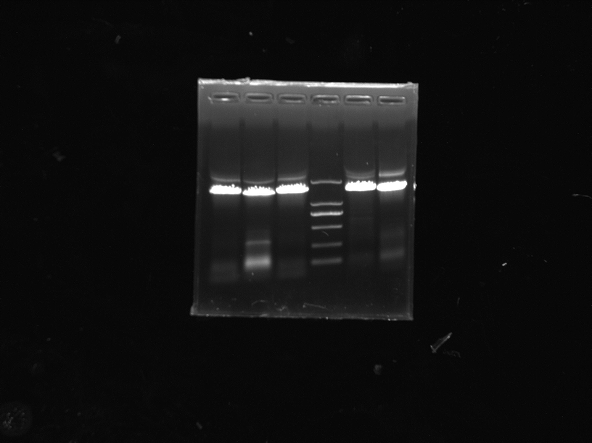

Supplement: S1 Fig — The raw image of Fig 1 in the manuscript is the S1 Fig legend. (TIF) [file pone.0285680.s001.tif]

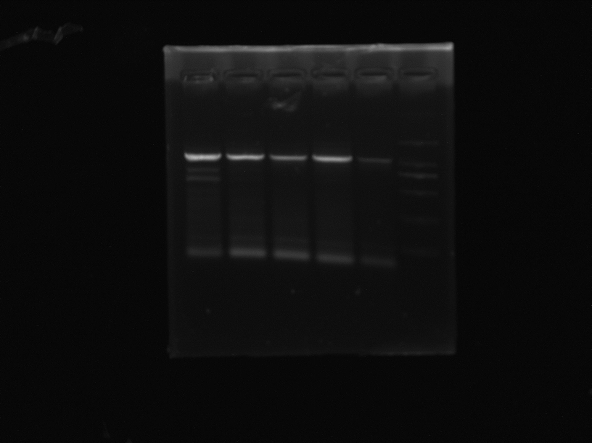

Supplement: S2 Fig — The raw image of Fig 1 is the S2 Fig legend. (TIF) [file pone.0285680.s002.tif]

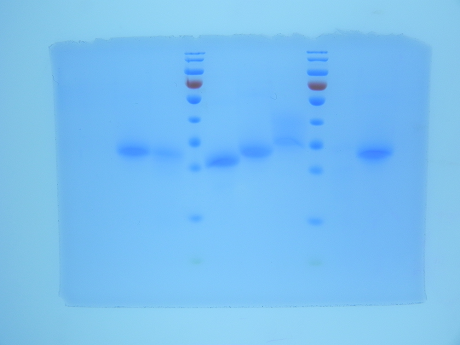

Supplement: S3 Fig — The raw image of Fig 3C in the manuscript is the S3 Fig legend. (TIF) [file pone.0285680.s003.tif]

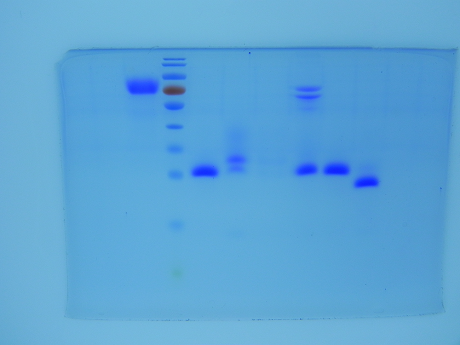

Supplement: S4 Fig — The raw image of Fig 3C in the manuscript is the S4 Fig legend. (TIF) [file pone.0285680.s004.tif]

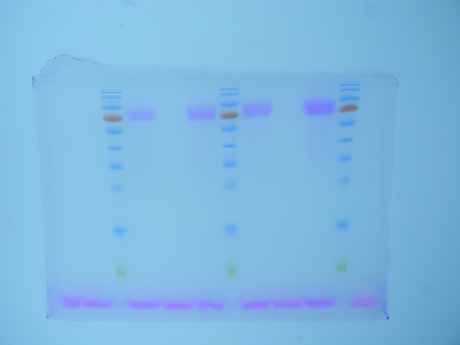

Supplement: S5 Fig — The raw image of Fig 4 in the manuscript is the S5 Fig legend. (TIF) [file pone.0285680.s005.tif]

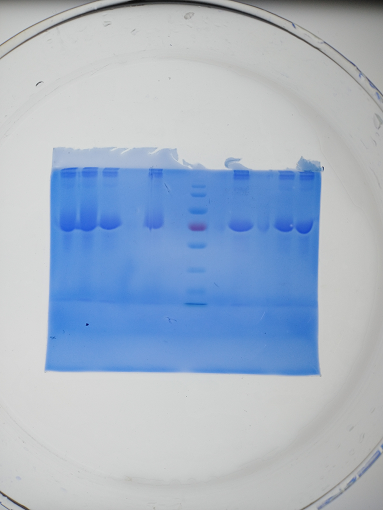

Supplement: S6 Fig — The raw image of Fig 5 in the manuscript is the S6 Fig legend. (TIF) [file pone.0285680.s006.tif]

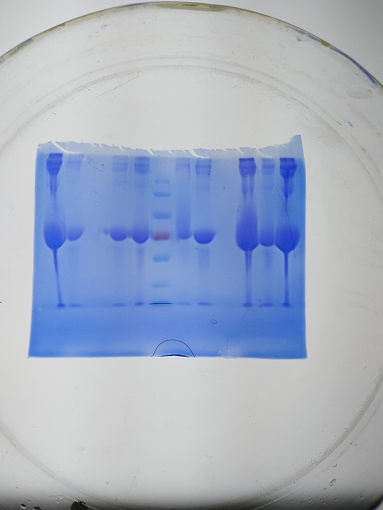

Supplement: S7 Fig — The raw image of Fig 5 in the manuscript is the S7 Fig legend. (TIF) [file pone.0285680.s007.tif]
