# Supplementary material for: Cloning, expression, and molecular modification of glycoside hydrolase family 5 genes from Thermoascus aurantiacus
Source: PLoS One. 2023 Sep 15;18(9):e0285680. doi: 10.1371/journal.pone.0285680 (PMC10503741; doi:10.1371/journal.pone.0285680)
Supplement: S1 File — (PDF) [file pone.0285680.s008.pdf]

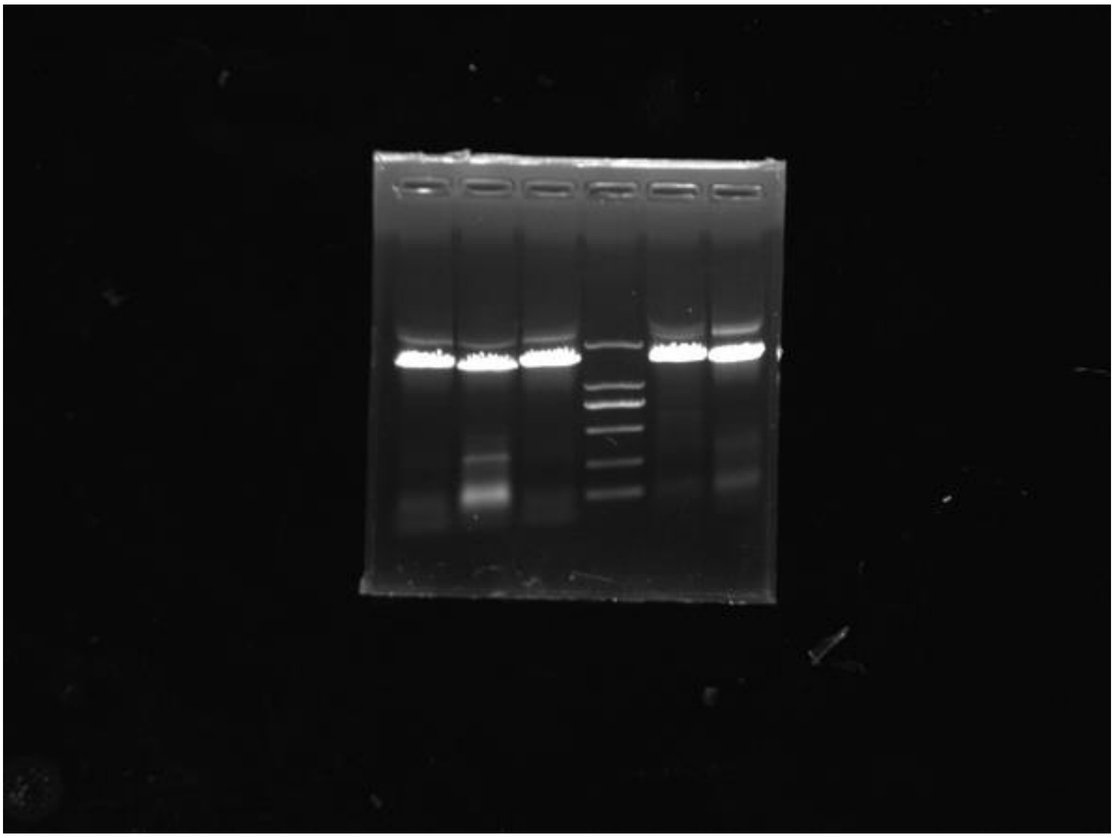

Figure1unprocessed version cel1

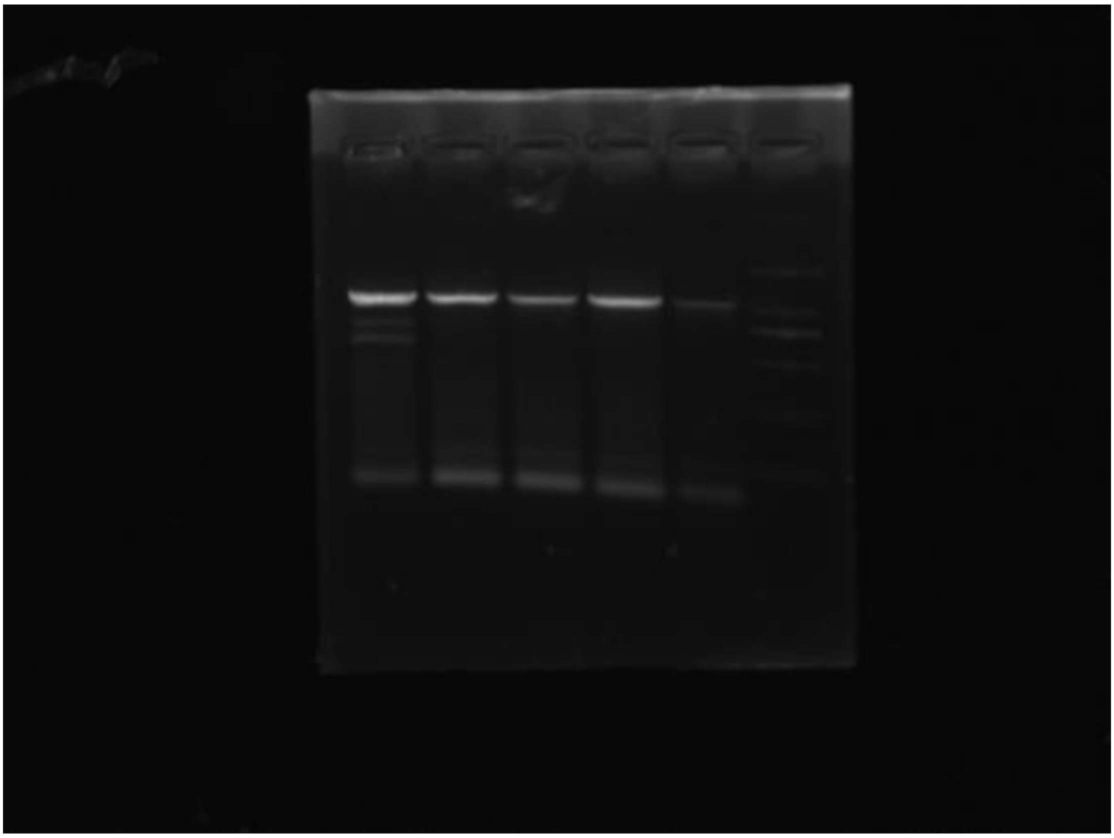

Figure1unprocessed version cel2

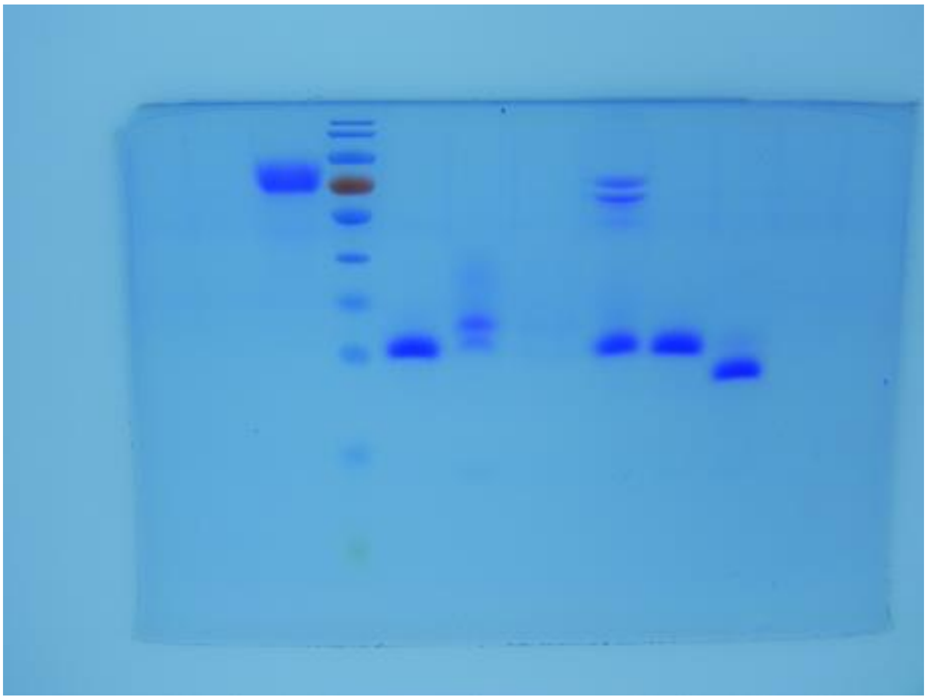

Fig.3unprocessed version1

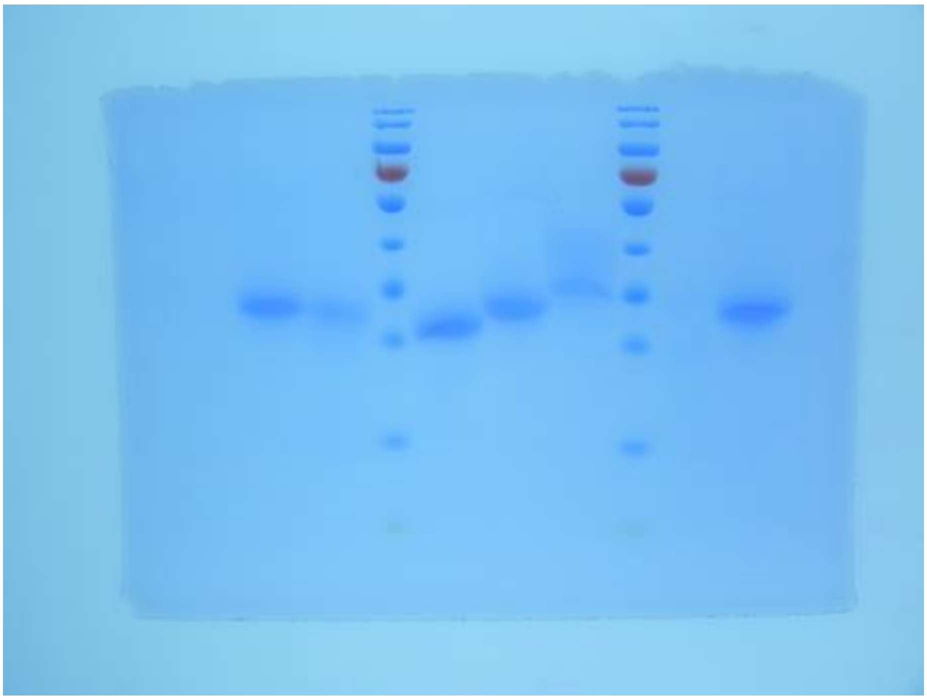

Fig.3unprocessed version2

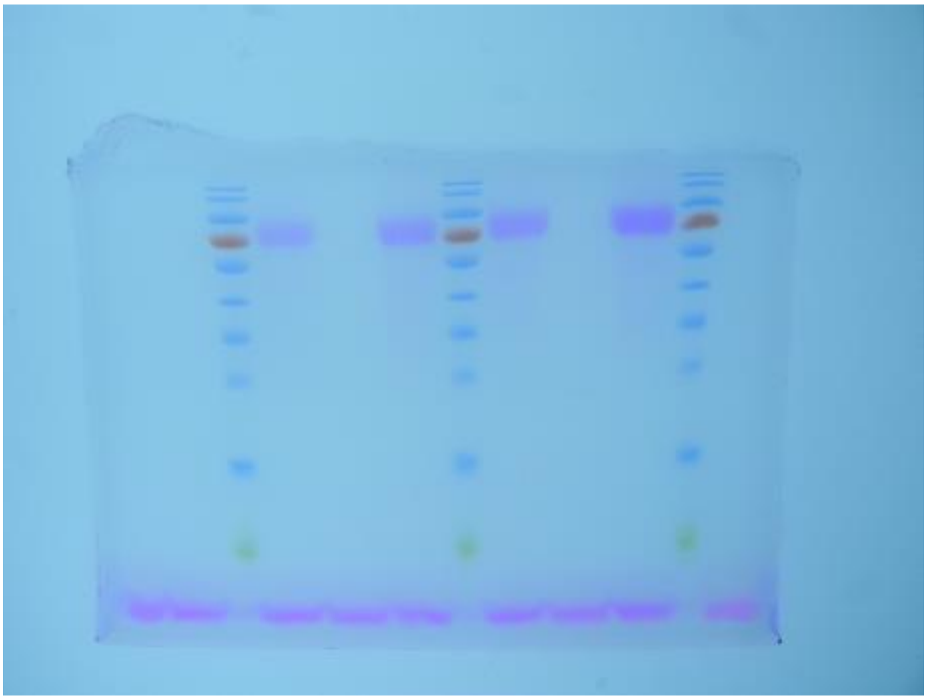
Fig.4unprocessed version
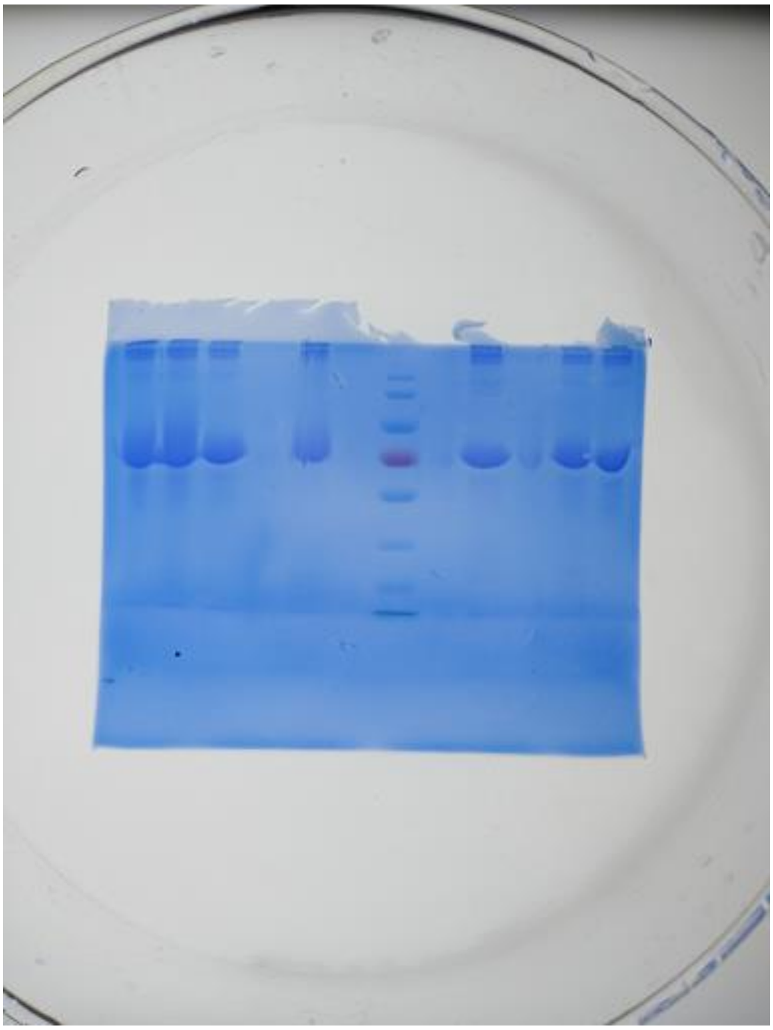
Fig.5unprocessed version1

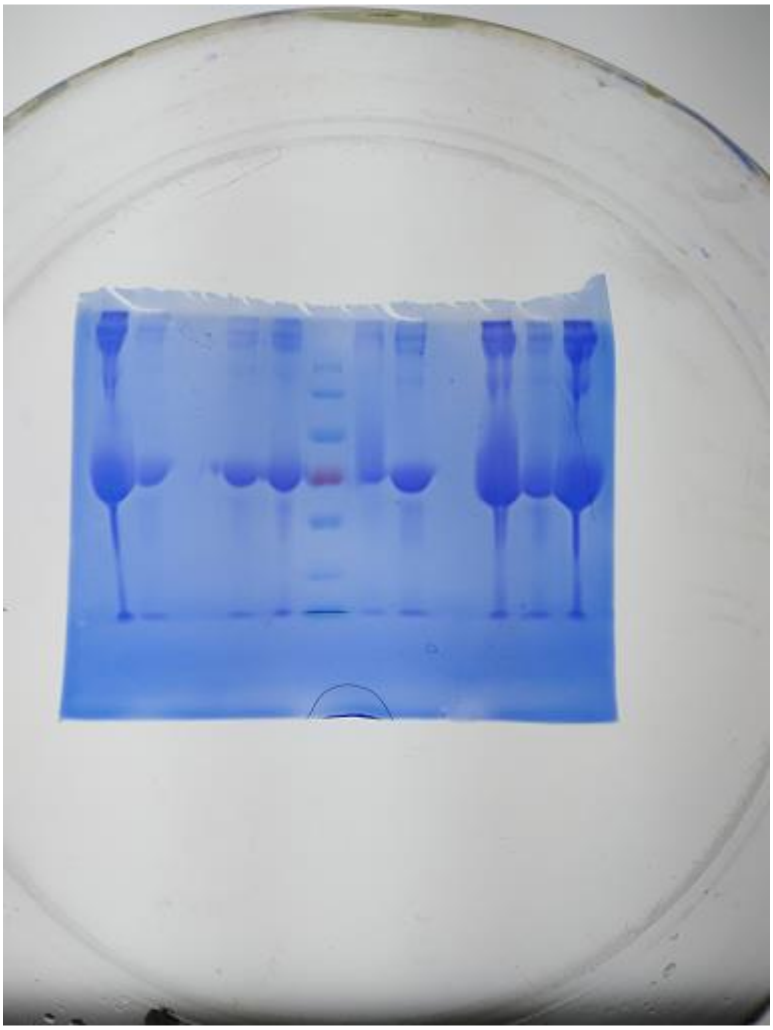

Fig.5unprocessed version2
