# Supplementary material for: Cloning, expression, and molecular modification of glycoside hydrolase family 5 genes from Thermoascus aurantiacus
Source: PLoS One. 2023 Sep 15;18(9):e0285680. doi: 10.1371/journal.pone.0285680 (PMC10503741; doi:10.1371/journal.pone.0285680)
Supplement: Attachment — Submitted filename: Response to Reviewers.doc [file pone.0285680.s009.doc]

Reviewer #1:

Line 89: the gene sequence of the cel1 gene (JC988729.1) was downloaded from GenBank. However, it is not clear if the gene sequence belongs to T. aurantiacus IMI strain.

Reply：

The gene sequence of the cel1 gene (JC988729.1) was downloaded from GenBank. The following information is available.SOURCE ：Thermoascus aurantiacus ORGANISM：Thermoascus aurantiacus Eukaryota; Fungi; Dikarya; Ascomycota; Pezizomycotina;Eurotiomycetes; Eurotiomycetidae; Eurotiales; Thermoascaceae;Thermoascus.

TITLE：POLYPEPTIDES HAVING ENDOGLUCANASE ACTIVITY AND POLYNUCLEOTIDES ENCODING SAME

JOURNAL：Patent: EP 2791329-A1 17 22-OCT-2014;NOVOZYMES INC [US]

Line 151 and Figure 3: The recombinant proteins were purified by either DEAE-Sepharose column or Purification by Ni-NTA affinity chromatography. Since the DEAE-Sepharose is a weak anion exchange chromatography resin, it is interesting to see that there was only one protein band on SDS-PAGE. Also, the purified protein band didn’t have degradation products. I recommend that the author revises figure 3 to include lanes of overexpression and purification procedure. Specifically, uninduced and induced P. pastoris GS115 and chromatography wash fraction.
Reply：

The recombinant proteins were purified by Ni-NTA affinity chromatography. There is only one protein band on SDS-PAGE.We can see similar results in several articles.For example, Cloning of a Gene Encoding β-Glucosidase from

Chaetomium thermophilum CT2 and Its Expression in Pichia pastoris. J Mol Microbiol Biotechnol 2011;20:16–23 DOI: 10.1159/000322606 .Figure 3 has been modified.Figure 3a and 3b were added.They are nickel column affinity chromatography for CEL1 and CEL2.Uninduced Pasteur's yeast GS115 was purified by Ni-NTA affinity chromatography.No single protein can be collected.

Figures 3 and 4: the nature of overexpressed Cel1 and Cel2 proteins is unknown. The purified proteins in Figure 3 and Figure 4 may be Cel1 and Cel2 proteins but could be something else. Additional assays, such as Western blot or mass spec, are needed to confirm that the purified proteins are Cel1 and Cel2 proteins.

Reply：

Six histidine tags were added to cel1 and cel2 clones.The genes cel1 and cel2 were expressed in yeastt GS115.The supernatant was applied to a Purification by Ni-NTA affinity chromatography(Figure3a、3b).The target protein can be obtained by collecting the crest part in Figure 3a、3b.The purified proteins in Figure 3c and Figure 4 are the Cel1 and Cel2 proteins.Uninduced Pasteur's yeast GS115 was purified by Ni-NTA affinity chromatography.No single protein can be collected.

Lines 159-160, Line 165, Line 194, and Line 203: The concentration of the recombinant protein was not stated in the assays since only 100 uL of the enzyme solution was stated.

Reply：

For example, comparing enzyme activity at different temperatures with the same enzyme solution.we don't have to list the concentration of the recombinant protein.On line 324, the concentration of the enzyme solution is listed.

Lines 82-82: The statement “the cDNA of T. aurantiacus was obtained by reverse
transcription polymerase chain reaction (RT-PCR )” is a little confusing. Please revise the purpose of doing RT-PCR.
Reply：

Modified.See lines 83, 84, 85

Lines 143-145: It was not clear what the inducer is for overexpression of the recombinant proteins.
Reply：

The recombinant protein was overexpressed as an inducer of methanol.See lines147

Lines 185-191: Did the assay have sample replicates in determining the Michaelis constant Km of the enzymes? Also, for Lines 212-218, Lines 220-226, and Lines 228-234: The same comment if the assays have sample replicates for determining the effects of different metal ions on enzyme activity, effects of different organic solvents on enzyme activity, and effects of salt tolerance.

Reply：

The experiment was repeated three times for the determination of the enzyme's Weil constant Km.Each experiment was repeated three times.The result accords with the requirement of data processing.

Lines 321-324: The manuscript reported that the CEL1 enzyme is not resistant to high temperatures above 65°C. However, another study reported that the enzyme from another strain of Thermoascus aurantiacus has an optimum temperature of 70°C.
Reply：

cel1 removes part of amino acid to obtain cel2. The change of structure may lead to the change of temperature resistance.The temperature curve was obtained according to the experimental results.

Line 339 and Figure 7: The author reported that the CEL1 enzyme’s activity was dramatically decreased when the pH is greater than pH5 and when the pH is lower than pH5. However, another study reported that the enzyme from another strain of Thermoascus aurantiacus is stable over a broad pH range of 3.0-7.0.

Reply：

cel1 removes part of amino acid to obtain cel2. The change of structure may lead to the change of pH resistance.The pH curve was obtained according to the experimental results.

Line 238: SPSS method was stated; however, the manuscript didn’t report any statistical results.

Reply：

Each experiment was repeated three times.The result accords with the requirement of data processing.The experimental curve was obtained according to the variance.See Figure 6、7、8、9、10。
